# Can the triglyceride-glucose index predict stroke outcomes? A systematic review and meta-analysis

**DOI:** 10.12669/pjms.41.10.12762

**Published:** 2025-10

**Authors:** Ying Zhou, Yusong Chen, Jiawei Wang

**Affiliations:** 1 Ying Zhou, Department of Rehabilitation Medicine, Changxing People’s Hospital, Huzhou, Zhejiang Province 313100, P.R. China; 2 Yusong Chen, Department of Rehabilitation Medicine, Changxing People’s Hospital, Huzhou, Zhejiang Province 313100, P.R. China; 3 Jiawei Wang, Department of Rehabilitation Medicine, Changxing People’s Hospital, Huzhou, Zhejiang Province 313100, P.R. China

**Keywords:** Triglyceride-glucose index, Stroke, Mortality, Stroke recurrence, Early neurological deterioration

## Abstract

**Objective:**

To assess the link between the triglyceride-glucose (TyG) index and stroke-related outcomes.

**Methodology:**

PubMed, Embase and Scopus databases were searched between January 1, 2000 to November 15, 2024 for studies reporting association between TyG and stroke outcomes. Pooled effect sizes were calculated as relative risks (RR) with 95% confidence intervals (CI).

**Results:**

Twenty five studies were included. High TyG levels significantly correlated with increased mortality during hospital stay (RR 1.70, 95% CI: 1.15 to 2.52), at three months (RR 1.96, 95% CI: 1.12 to 3.45) and at 12 months (RR 1.43, 95% CI: 1.07 to 1.91) follow-ups. Similarly, high TyG levels were linked to higher recurrence risk at three months (RR 2.75, 95% CI: 1.31 to 5.78) and 12 months (RR 1.41, 95% CI: 1.18 to 1.68) post-stroke. Elevated TyG was also linked to an increased risk of early neurological deterioration (END) during hospital stay (RR 3.28, 95% CI: 1.71 to 6.30) and poor functional outcome at three months (RR 1.67, 95% CI: 1.18 to 2.37) follow-up. Subgroup analyses showed consistent results across study designs, geographic locations and sample sizes. Low to very low certainty of evidence across various outcomes was detected by GRADE.

**Conclusions:**

Elevated TyG index might be a significant predictor of mortality, recurrence, END and poor functional outcomes in stroke patients, emphasizing its potential as a prognostic biomarker. Further studies are needed to explore its utility in clinical decision-making.

***Registration No.:*** PROSPERO CRD42024613240.

## INTRODUCTION

Stroke is responsible for considerable mortality and disability globally.^1^ Ischemic stroke (IS) accounts for most of stroke cases (nearly 65%).^1^ While stroke predominantly affects the elderly, there is a gradual increase in the prevalence of stroke in younger adults.^2^ Stroke not only leads to physical and cognitive disabilities but also contributes to a substantial burden on healthcare systems and on the patients and their families due to loss of productivity and high treatment costs.^3^

Despite significant advancements in acute management and preventive strategies, stroke recurrence remains a persistent challenge.^4^,^5^ This high recurrence rate underscores the need for improved risk stratification and secondary prevention strategies. Insulin resistance (IR) is increasingly recognized as pivotal in stroke pathophysiology.^6^,^7^ The triglyceride-glucose (TyG) index is an easy-to-use and affordable marker of insulin resistance (IR). It has gained attention for its potential utility in predicting cardiovascular and cerebrovascular outcomes.^8^-^10^ The index utilizes fasting triglyceride and glucose levels. It offers a feasible alternative to the hyperinsulinemic-euglycemic clamp (HIEC), which is resource-demanding and challenging to implement in clinical settings.^11^,^12^

A meta-analysis by Yang Y et al. revealed a significant correlation of elevated TyG index and a higher risk of stroke recurrence and mortality in IS patients. In contrast, no significant associations were reported between the TyG index and poor functional outcomes or neurological worsening.^13^ This review did not include studies involving patients with hemorrhagic stroke and only included studies published up to April 2022. Considering the significant number of studies published on this topic over the past two and a half years, an updated and more comprehensive meta-analysis was necessary. The current meta-analysis aims to investigate the association of the TyG index with stroke outcomes, i.e., mortality, poor functional recovery, early neurological deterioration (END) and recurrence.

## METHODOLOGY

This meta-analysis followed the PRISMA guidelines and registered on PROSPERO (CRD42024613240).^14^

### Inclusion criteria:

- Adult patients (≥18) with ischemic or hemorrhagic stroke diagnoses.
- The TyG index assessed specifically at the time of admission for the stroke event.
- Studies reporting one of the following outcomes: stroke recurrence, mortality, poor functional outcomes and END.
- Studies with confounder-adjusted estimates.
- Studies with the sample size > 100. This was done to exclude studies with very small sample size and avoid small-study bias.

### Exclusion criteria:

- Studies without a clear or confirmed diagnosis of stroke.
- Studies that included subjects with pre-existing neurological disorders that could confound the results.
- Case reports, reviews, editorials and commentaries.

### Search strategy:

PubMed, Embase and Scopus databases were searched for English language studies (Supplementary Table-I). The search was restricted to studies published between January 1, 2000 to November 15, 2024. Additionally, manual searches of bibliographies were conducted to ensure that all pertinent studies were identified, complementing the electronic search process. Following the implementation of the search strategy across the databases, duplicate studies were removed electronically as the initial step. Titles and abstracts were systematically screened by the two independent reviewers (YZ, YC), followed by a full-text assessment of the potentially relevant articles. Any disagreements regarding study inclusion were addressed through discussions between the authors.

**Supplementary Table-I:** Search terms used in different databases.

***Pubmed***
(("stroke"[MeSH Terms] OR "ischemic stroke"[Title/Abstract] OR "hemorrhagic stroke"[Title/Abstract])
AND ("triglyceride-glucose index"[Title/Abstract] OR "TyG index"[Title/Abstract] OR "triglyceride glucose"[Title/Abstract])
AND ("mortality"[MeSH Terms] OR "functional outcomes"[Title/Abstract] OR "neurological worsening"[Title/Abstract] OR "recurrence"[Title/Abstract]))
AND ("humans"[MeSH Terms])
AND ("english"[Language])
AND ("2000/01/01"[Date - Publication] : "2024/11/15"[Date - Publication])

***Embase***
((’stroke’/exp OR ’ischemic stroke’:ti,ab OR ’hemorrhagic stroke’:ti,ab)
AND (’triglyceride glucose index’:ti,ab OR ’TyG index’:ti,ab OR ’triglyceride glucose’:ti,ab))
AND (’mortality’/exp OR ’functional outcome’:ti,ab OR ’neurological worsening’:ti,ab OR ’recurrence’:ti,ab)
AND [humans]/lim
AND [english]/lim
AND [2000-2024]/py

***Scopus***
(TITLE-ABS-KEY("stroke" OR "ischemic stroke" OR "haemorrhagic stroke" OR "hemorrhagic stroke")
AND TITLE-ABS-KEY("triglyceride glucose index" OR "TyG index" OR "triglyceride glucose")
AND TITLE-ABS-KEY("mortality" OR "functional outcome" OR "neurological worsening" OR "recurrence"))
AND (LIMIT-TO(LANGUAGE, "English"))
AND (LIMIT-TO(DOCTYPE, "ar"))
AND (PUBYEAR > 1999 AND PUBYEAR < 2025)

### Data extraction and quality assessment:

Data were extracted independently by the two authors (YZ, JW) using a standardized form that included study identifiers (e.g., first author along with the year of publication), study design and location, age and sex distribution of participants, cut-off of TyG used, type of stroke, sample size, factors adjusted in the analytic mode, time of outcome assessment and findings on key outcomes measured. Any discrepancies between the two authors were discussed to reach a consensus. The risk of bias was assessed by the Newcastle-Ottawa Scale (NOS). The scores ranged from 0-9. Studies with score of 8-9 were considered high, 6-7, medium and <6 as low quality. Quality assessment was conducted independently by two reviewers.^15^

### Statistical analysis:

The pooled effect sizes were calculated as relative risks (RR) and reported with 95% confidence intervals (CI). A random-effects model was employed.^16^ Subgroup analysis was conducted for mortality at 12 months post-stroke, END (in-hospital), poor functional outcome (at three and 12 months after the stroke) and recurrence (at 12 months post-stroke). Subgroup analyses were performed only for time points where more than three studies contributed to the outcome. Similarly, publication bias was evaluated by Egger’s test and funnel plots but only for time points reported by more than three studies.^17^,^18^ Inter-study heterogeneity was assessed using Cochrane’s I^2^ statistic with values >50% indicating high heterogeneity. P<0.05 was considered significant. Subgroup analyses were stratified based on study design, geographic location and sample size.

## RESULTS

**Fig.1:**
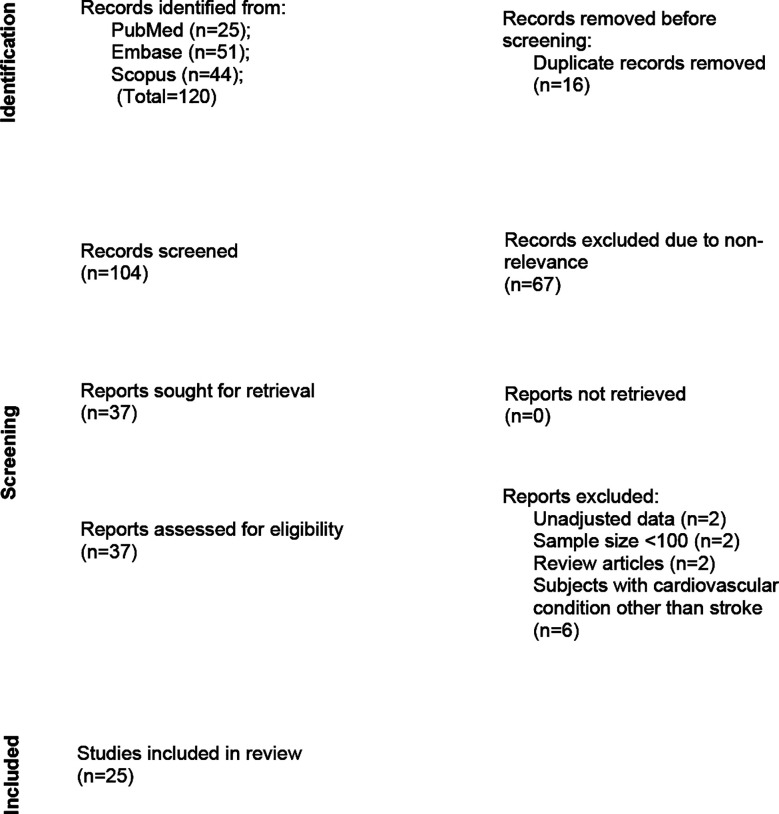
Selection process of studies included in the review.

The systematic search identified 120 studies. After screening, 25 studies were included (Fig.1).^19^-^43^ The included studies had 35,953 participants (Supplementary Table-II). Most studies focused on participants with IS. One study included subjects with hemorrhagic stroke, while another had patients with either ischemic or hemorrhagic stroke. There was considerable variation in how TyG levels were analyzed in relation to stroke outcomes. Most studies stratified TyG levels into quartiles or tertiles while a smaller number treated TyG as a binary variable (high/low based on a cut-off) or as a continuous variable. Definitions of outcomes varied across studies. For example, some studies defined poor functional outcomes as a modified Rankin Score (mRS) of 3-6, whereas others used a score of 4-6. NOS assessment showed that the quality of included studies had a mean score of 8.0 (Supplementary Table-III).

**Supplementary Table-II:** Summary of the studies included in the meta-analysis.

Study identifier	Design and location	Cut-off used for TyG ratio	Type of stroke	Age (years) and Males (%)	Sample size	Time of outcome assessment	Outcome assessment	NOS score
Cheng Y et al. (2024)^19^	Cohort (P); China	Tertile based; T3, (9.13≤TyG index≤11.43) compared to T1 (7.27≤TyG index≤8.58)	Ischemic	68; 62%	210	Three months post-stroke	Cognitive impairment: The Montreal Cognitive Assessment (MoCA) score of *<* 26 suggested impairments	7
Wang J et al. (2024)^20^	Cohort (R); China	Tertile based; T3, (≥9.486 mg/dl) compared to T1, (<8.80 mg/dl)	Ischemic	61 ; 66%	1696	At the time of hospital stay	END: increase of ≥ 2 points in the patient’s NIHSS score within one week of admission compared with the admission	8
Guo W et al. (2024)^21^	Cohort (R); China	Quartile based; Q4, ( ≥9.13 mg/dl) compared to Q1, (<8.31 mg/dl)	Ischemic	64 ; 63%	1144	12 months	Recurrence of stroke: diagnosed by neurologists (basis of neurological and radiological findings)	8
Deng M et al. (2024)^22^	Cohort (R); China	Binary cut-off with high TyG defined as value ≥7.17 mg/dl	Ischemic	66 ; 62%	1187	At the time of hospital stay	END: increase in the NIHSS score by ≥4 points in the total score within 24 h after thrombolysis	9
Liu H et al. (2024)^23^	Cohort (R); China	Used as a continuous variable	Ischemic	71 ; 57%	412	At three months post-discharge	Poor functional outcome: Modified Rankin Scale (mRS) Score of 3 to 6	8
Huang Y et al. (2024)^24^	Cohort (R); China	Quartile based; Q4, (9.2 to 12.2 mg/dl) compared to Q1, (7.1 to 8.4 mg/dl)	Haemorrhagic	69 ; 54%	1475	At 1-month post-discharge	---	9
Miao M et al. (2023)^25^	Cohort (R); China	Quartile based; Q4, ( ≥9.12 mg/dl) compared to Q1, (<8.25 mg/dl)	Ischemic	69 ; 58%	1608	At time of hospital stay	Poor functional outcome: Modified Rankin Scale (mRS) Score of 4 to 6	8
Wang Y et al. (2023)^26^	Cohort (P); China	Quartile based; Q4, ( ≥9.4 mg/dl) compared to Q1, (<8.4 mg/dl)	Ischemic	62 ; 70%	641	three months	Recurrence: acute new focal neurologic deficit lasting for more than 24 h, with an increase in the NIHSS score of four or more, or imaging evidence on MRI or CT	7
Cai W et al. (2023)^27^	Cohort (R); China	Quartile based; Q4, (9.34 to 11.34 mg/dl) compared to Q1, (7.29 to 8.53 mg/dl)	Ischemic	69 ; 55%	367	At the time of hospital stay	---	8
Wang J et al. (2023)^28^	Cohort (R); China	Tertile based; T3, (≥9.40 mg/dl) compared to T1, (<8.66 mg/dl)	Ischemic	60 ; 63%	2129	At the time of hospital stay	END: deterioration of ≥ 2 points in the patient’s NIHSS score within 7 days of admission compared with the admission	8
Liu R et al. (2023)^29^	Cohort (R); China	Tertile based; T3, (9.04 to 12.9 mg/dl) compared to T1, (5.91 to 8.50 mg/dl)	Ischemic	50 ; 65%	1226	3 and 12 months	Poor functional outcome: Modified Rankin Scale (mRS) Score of 3 to 6	9
Zhang B et al. (2023)^30^	Cohort (R); China	Tertile based; the specific cut-off values within each three strata not specified	Ischemic	68 ; 64%	449	At the time of hospital stay	END: increase in the NIHSS score by ≥2 points in the total score within 24 h after thrombolysis	8
Tutal GG et al. (2023)^31^	Cohort (R); Turkey	Used as a continuous variable	Ischemic	67 ; 49%	147	three months post-discharge	Poor functional outcome: Modified Rankin Scale (mRS) Score of 3 to 6	7
Wu L et al. (2022) 32	Cohort (P); China	Quartile based; Q4, (>8.92 mg/dl) compared to Q1, (<8.20 mg/dl)	Ischemic	66 ; 68%	986	12 months	Recurrence of stroke: based on appearance of new neurological symptoms and verified by MRI	9
Hoshino T et al. (2022) 33	Cohort (P); Japan	Tertile based; T3, (>9.01 mg/dl) compared to T1, (<8.48 mg/dl)	Ischemic (90%)	70 ; 61%	587	12 months	Recurrence of stroke: diagnosed by board-certified stroke neurologists (on basis of neurological and radiological findings)	8
Liu D et al. (2022) (34)	Cohort (R); China	Quartile based; Mean (SD) TyG in Q4 was 2.9 (0.4) mmol/L and in Q1 was 1.0 (0.6) mmol/L	Ischemic	65 ; 59%	1697	12 months	Poor functional outcome: mRS. score of 3 to 6	9
Yang X et al. (2022) 35	Cohort (R); China	Quartile based; Q4, (6.22 to 8.17 mg/dl) compared to Q1, (4.31 to 5.48 mg/dl)	Ischemic	62 ; 65%	612	12 months post-discharge	Recurrence: defined as an aggravated primary neurological deficit, a new neurological deficit, or re-hospitalization caused by ischemic or hemorrhagic stroke	8
Lin SF et al. (2022) 36	Cohort (P); Taiwan	Tertile based; T3, (>9.04 mg/dl) compared to T1, (<8.48 mg/dl)	Ischemic	69 ; 64%	914	At three months post-discharge	Poor functional outcome: Modified Rankin Scale (mRS) Score of 3 to 6	7
Toh EMS et al. (2022) 37	Cohort (R); Singapore	Binary cut-off with high TyG defined as value ≥9.15 mg/dl	Ischemic	65 ; 62%	698	At three months post-discharge	END: increase in NIHSS score by ≥4 points within 24 hour Poor functional outcome: Modified Rankin Scale (mRS) Score of 3 to 6	8
Lee M et al. (2021)^38^	Cohort (R); Republic of Korea	Binary cut-off with high TyG defined as value ≥4.49 mg/dl	Ischemic	70 ; 59%	183	At three months post-discharge	Poor functional outcome: Modified Rankin Scale (mRS) Score of 3 to 6	7
Nam KW (A) et al. (2021)^39^	Cohort (R); Republic of Korea	Used as a continuous variable	Ischemic	68 ; 59%	305	At the time of hospital stay	END: increase of ≥2 in the total NIHSS score or ≥1 in the motor NIHSS score compared to initial NIHSS score within the first 72 h of admission	7
Hou Z et al. (2021)^40^	Cohort (R); China	Quartile based; Q4, (>9.21 mg/dl) compared to Q1, (<8.33 mg/dl)	Ischemic	64 ; 65%	6483	12 months post-discharge	Poor functional outcome: Modified Rankin Scale (mRS). Score of 3 to 6 Recurrence: Research staff verified stroke recurrences through hospital records and adjudicated non-hospitalized cases	9
Nam KW (B) et al. (2021)^41^	Cohort (R); Republic of Korea	Tertile based; T3, (>8.63 mg/dl) compared to T1, (<8.24 mg/dl)	Ischemic	70 ; 58%	176	At the time of hospital stay	Recurrence: based on new diffusion-weighted imaging lesions outside the initial symptomatic lesion area	8
Zhou Y et al. (2020)^42^	Cohort (R); China	Quartile based; Q4, (≥9.21 mg/dl) compared to Q1, (≤8.33 mg/dl)	Ischemic	65 ; 64%	8155	12 months post-discharge END: assessed during the time of hospital stay	END: ≥2-point increase in NIHSS score during hospitalization compared with the score on admission	8
Zhang B et al. (2020)^43^	Cohort (R); China	Quartile based; Q4, (mean 10 ±0.1 mg/dl) compared to Q1, (mean 8.3±0.3 mg/dl)	Mix of ischemic (~44%) and haemorrhagic stroke (~30%)	66 ; 51%	2466	At time of hospital stay	---	8

**Supplementary Table-III:** Risk of bias analysis.

Study identifier	Selection	Comparability	Outcome assessment	NOS score
Cheng Y et al. (2024)^19^	4	2	1	7
Wang J et al. (2024)^20^	4	2	2	8
Guo W et al. (2024)^21^	4	2	2	8
Deng M et al. (2024)^22^	4	2	2	8
Liu H et al. (2024)^23^	4	2	2	8
Huang Y et al. (2024)^24^	4	2	3	9
Miao M et al. (2023)^25^	4	2	2	8
Wang Y et al. (2023)^26^	4	2	1	7
Cai W et al. (2023)^27^	4	2	2	8
Wang J et al. (2023)^28^	4	2	2	8
Liu R et al. (2023)^29^	4	2	3	9
Zhang B et al. (2023)^30^	4	2	2	8
Tutal GG et al. (2023)^31^	4	2	1	7
Wu L et al. (2022)^32^	4	2	3	9
Hoshino T et al. (2022)^33^	4	2	2	8
Liu D et al. (2022)^34^	4	2	3	9
Yang X et al. (2022)^35^	4	2	2	8
Lin SF et al. (2022)^36^	4	2	1	7
Toh EMS et al. (2022)^37^	4	2	2	8
Lee M et al. (2021)^38^	4	2	1	7
Nam KW (A) et al. (2021)^39^	4	2	1	7
Hou Z et al. (2021)^40^	4	2	3	9
Nam KW (B) et al. (2021)^41^	4	2	2	8
Zhou Y et al. (2020)^42^	4	2	2	8
Zhang B et al. (2020)^43^	4	2	2	8

### Risk of mortality:

**Fig.2:**
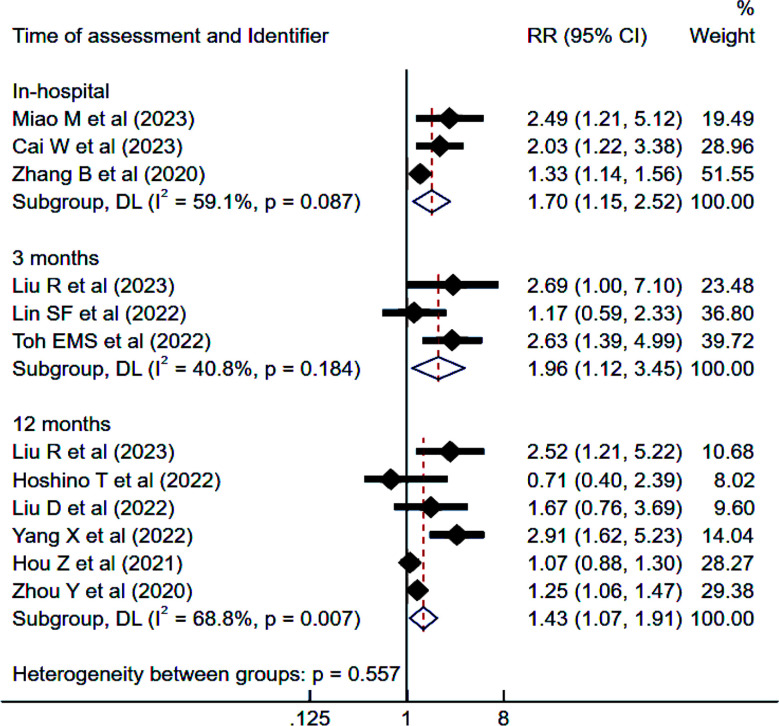
of mortality among those with high triglyceride-glucose (TyG) index compared to those with low TyG, in subjects with ischemic stroke. RR, risk ratio; CI, confidence intervals.

IS patients with elevated TyG had higher risk of mortality during in-hospital stay (RR 1.70, 95% CI: 1.15 to 2.52; I^2^=59.1%), at three months (RR 1.96, 95% CI: 1.12 to 3.45; I^2^=40.8%) and 12 months (RR 1.43, 95% CI: 1.07 to 1.91; I^2^=68.8%) of follow-up compared to patients with low TyG (Fig.2). There was no evidence of publication bias (Egger’s p-value 0.32). Each unit increase in TyG in IS patients was also associated with elevated risk of mortality at each time point i.e., in-hospital (RR 1.36, 95% CI: 1.19 to 1.55; I^2^=0.0%), three months (RR 1.69, 95% CI: 1.05 to 2.74; I^2^=57.3%) and 12 months (RR 1.12, 95% CI: 1.02 to 1.22) of follow-up.

In patients with hemorrhagic stroke, high TyG levels were linked to an increased risk of mortality at one month of follow-up compared to low TyG levels (RR 1.22, 95% CI: 0.95 to 1.57; I²=40.0%). Additionally, for each unit increase in the TyG index in hemorrhagic stroke patients, the risk of mortality at one month of follow-up also increased (RR 1.29, 95% CI: 1.08 to 1.53; I²=0%).

The subgroup analysis demonstrated a considerable association between high TyG levels and increased mortality at 12 months follow-up in retrospective studies, studies with ≥500 patients and studies conducted in China (Supplementary Table-IV).

**Supplementary Table-IV:** Findings of the subgroup analysis.

	Mortality (12 months)	END (In-hospital)	Poor functional outcome (three months)	Poor functional outcome (12 months)	Recurrence (12 months)
	***Relative risk (RR) (95% confidence interval) (Number of studies; I^2^)***				
***Study design***					
Prospective	0.71 (0.29, 1.74) (N=1)	---	1.76 (1.13, 2.75) (N=1)	---	2.23 (1.36, 3.67) (N=2; 0.0)
Retrospective	1.52 (1.12, 2.05) (N=5; 72.4)	3.28 (1.71, 6.30) (N=5; 95.6)	1.73 (1.02, 2.94) (N=3; 63.4)	1.00 (0.83, 1.19) (N=4; 57.7)	1.30 (1.15, 1.47) (N=5; 3.0)
***Sample size***					
≥500	1.43 (1.07, 1.91) (N=6; 68.8)	3.40 (1.59, 7.27) (N=4; 96.6)	1.53 (1.17, 1.99) (N=3; 21.1)	1.00 (0.83, 1.19) (N=4; 57.7)	1.41 (1.18, 1.68) (N=7; 35.0)
<500	---	2.82 (1.61, 4.94) (N=1)	5.22 (1.39, 19.6) (N=1)	---	---
***Study location***					
China	1.52 (1.12, 2.05) (N=5; 72.4)	3.28 (1.71, 6.30) (N=5; 95.6)	1.23 (0.87, 1.73) (N=1)	1.00 (0.83, 1.19) (N=4; 57.7)	1.37 (1.14, 1.64) (N=6; 35.8)
Outside China	0.71 (0.29, 1.74) (N=1)	---	1.93 (1.36, 2.74) (N=3; 16.0)	---	1.92 (1.05, 3.50) (N=1)

### Risk of recurrence:

**Fig.3:**
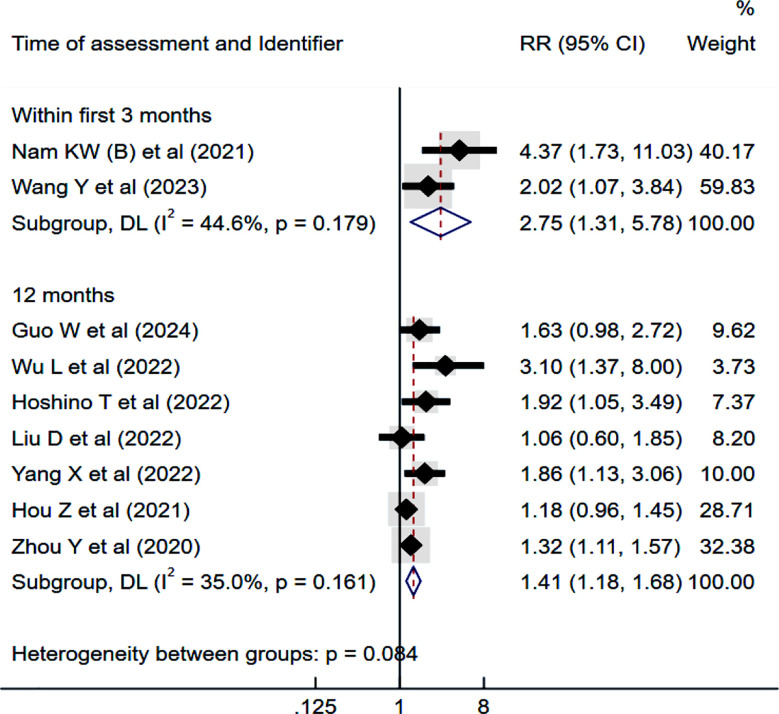
Risk of recurrence among those with high triglyceride-glucose (TyG) index compared to those with low TyG, in subjects with ischemic stroke. RR, risk ratio; CI, confidence intervals.

IS patients with high TyG had an increased risk of recurrence within the first three months of follow-up (RR 2.75, 95% CI: 1.31 to 5.78; I^2^=44.6%) and at 12 months follow-up (RR 1.41, 95% CI: 1.18 to 1.68; I^2^=35.0%) (Fig.3). No publication bias was detected (Egger’s p-value 0.10). Each unit increase in TyG was not significantly associated with the risk of recurrence within the initial three months of follow-up (RR 1.13, 95% CI: 0.85 to 1.50). However, at 12 months of follow-up, each unit increase in TyG was associated with a higher risk of recurrence (RR 1.40, 95% CI: 1.00 to 1.96; I²=72.6%).

In the subgroup analysis, both prospective and retrospective studies demonstrated an increased risk of recurrence associated with high TyG levels. Larger studies (sample size ≥500), as well as studies conducted in China or outside China, also reported significant associations.

### Risk of END:

**Fig.4:**
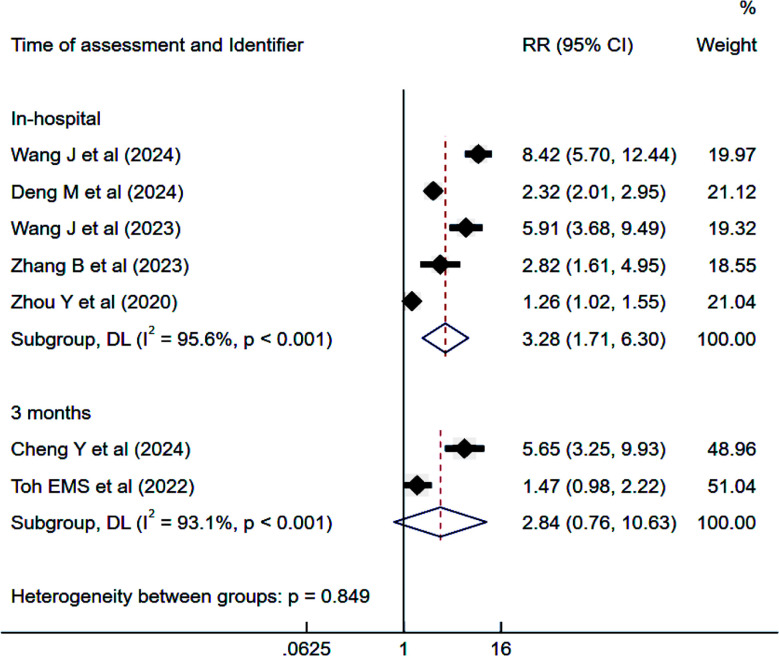
Risk of early neurological deterioration among those with high triglyceride-glucose (TyG) index compared to those with low TyG, in subjects with ischemic stroke. RR, risk ratio; CI, confidence intervals.

IS patients with high TyG exhibited an increased risk of END at the time of hospital stay (RR 3.28, 95% CI: 1.71 to 6.30; I^2^=95.6%) but not at three months follow-up (RR 2.84, 95% CI: 0.76 to 10.6; I^2^=93.1%) (Fig.4). No evidence of publication bias was detected (Egger’s p-value 0.21). Each unit increase in TyG was significantly associated with an elevated risk of END during hospitalization (RR 1.90, 95% CI: 1.44 to 2.52; I²=95.4%) and at three months of follow-up (RR 1.47, 95% CI: 1.12 to 1.92).

In the subgroup analysis, retrospective studies reported a significant link between high TyG levels and an elevated risk of in-hospital END. Studies with larger sample sizes (≥500) demonstrated a higher magnitude of risk compared to smaller studies.

### Risk of poor functional outcome:

**Fig.5:**
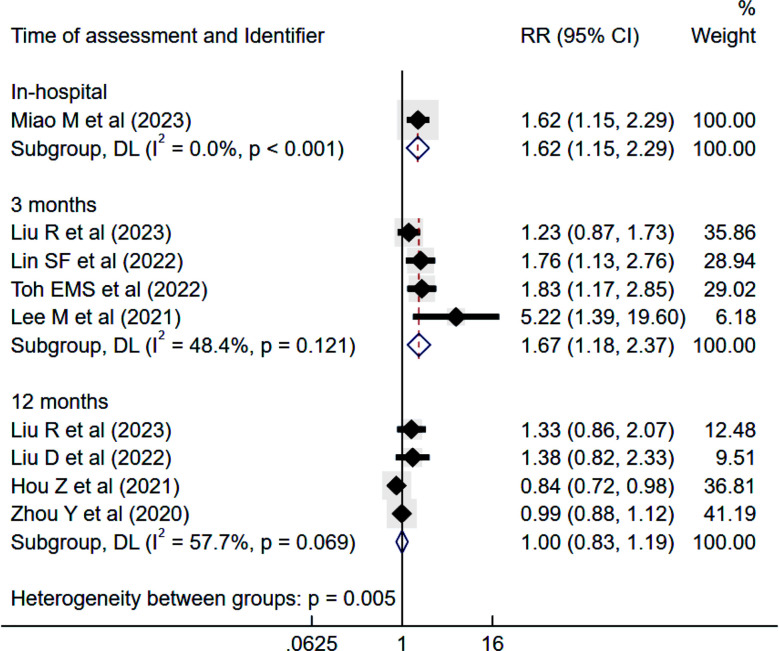
Risk of poor functional outcome among those with high triglyceride-glucose (TyG) index compared to those with low TyG, in subjects with ischemic stroke. RR, risk ratio; CI, confidence intervals.

IS patients with high TyG had higher risk of PFO during hospital stay (RR 1.62, 95% CI: 1.15 to 2.29) and at three months (RR 1.67, 95% CI: 1.18 to 2.37; I^2^=48.4%) follow-up but not at 12 months of follow-up (RR 1.00, 95% CI: 0.83 to 1.19; I^2^=57.7%) (Fig.5).

No evidence of publication bias was detected (Egger’s p-value 0.09). Each unit increase in TyG was associated with the increased risk of PFO during hospital stay (RR 1.18, 95% CI: 1.05 to 1.32) and at three months of follow-up (RR 1.59, 95% CI: 1.14 to 2.21; I^2^=47.8%) but not at 12 months of follow-up (RR 1.00, 95% CI: 0.94 to 1.06). The subgroup analysis showed that at three months, both prospective and retrospective studies demonstrated a significant association between elevated TyG and poor functional outcomes (Supplementary Table-IV). Similarly, a significant association was observed regardless of the sample size.

## DISCUSSION

This meta-analysis demonstrates that high TyG levels were consistently linked to increased risks of mortality, stroke recurrence, END and poor functional outcomes across various time points and subgroups. While most outcomes were reported for the IS survivors, the findings related to increased risk of short-term mortality also extend to hemorrhagic stroke, reinforcing the relevance of TyG as a prognostic marker. High TyG levels correlated with increased risks of mortality during hospitalization and up to 12 months post-stroke, higher recurrence rates at three and 12 months, elevated risks of END during hospitalization and poor functional outcomes at three months. These associations were more pronounced in retrospective studies, studies with larger sample sizes and those conducted in China. From a clinical perspective, the TyG index could be considered as a cost-effective biomarker for early risk stratification and long-term monitoring in stroke patients. However, its inconsistent ways of classifying high and low TyG levels and varied measurement methods for the outcomes across studies necessitate standardization to enhance its clinical applicability.

This study represents a more complete analysis of the link between the TyG index and stroke outcomes compared to the previous review by Yang Y et al.^13^ The earlier review, which included 10 studies, did not specify if adjusted estimates were used in the pooled analysis and lacked clarity on follow-up time points for reported outcomes. While Yang et al. found that a higher TyG index correlated with increased stroke recurrence and mortality in IS patients, no significant associations were noted for poor functional outcomes or neurological worsening.^13^ In contrast, the current review demonstrates that elevated TyG is linked to a higher END risk and poor functional outcomes. Our review offers a more robust and up-to-date assessment by incorporating more recent studies, adjusting for confounders and providing outcome estimates across various follow-up periods.

Some underlying biological mechanisms may explain the observed associations between elevated TyG levels and adverse stroke outcomes. The TyG index, a marker of insulin resistance, reflects the complex interplay between dysregulated glucose and lipid metabolism.^44^ Studies show that insulin resistance promotes endothelial dysfunction, increases vascular inflammation and impairs nitric oxide production, compromising cerebrovascular integrity and function.^45^,^46^ These pathophysiological changes increase the risk of ischemic events, worsen stroke severity and impede recovery processes, potentially contributing to poor functional outcomes and higher mortality.^47^-^49^ Hyperglycemia, a hallmark of insulin resistance, may exacerbate ischemic injury by amplifying oxidative stress.^50^,^51^ Excess glucose levels can also enhance the generation of advanced glycation end products, which in turn activate inflammatory pathways and cause endothelial damage.^52^,^53^

Concurrently, elevated triglyceride levels are associated with the formation of small, dense low-density lipoprotein particles, which are highly atherogenic.^54^ This atherogenesis ultimately increases the risk of stroke recurrence.^54^,^55^ Insulin resistance and dyslipidemia are closely associated with systemic inflammation with elevated levels of pro-inflammatory interleukin-6 and tumor necrosis factor-alpha. These cytokines not only exacerbate atherosclerosis but also disrupt the blood-brain barrier, intensifying the severity of stroke and hindering recovery.^56^,^57^

Growing evidence supports the link between TyG and cardiovascular and cerebrovascular conditions. Studies show that elevated TyG levels are linked to a bigger risk of CAD, peripheral artery disease and infarction.^8^-^10^ Elevated TyG correlate with greater plaque instability, elevated risk of adverse cardiovascular events and higher risk of carotid atherosclerosis and intracranial arterial stenosis, suggesting its involvement in subclinical vascular pathology.^13^ Furthermore, TyG has been linked to the onset of heart failure, atrial fibrillation and microvascular complications.^8^-^10^

### Limitations:

First, the predominance of retrospective studies may have introduced selection and recall biases. Second, the heterogeneity in TyG stratification and outcome definitions across studies may have influenced the pooled effect estimates. Third, the geographic skew towards studies conducted in China restricts the applicability of the findings. Fourth, the relatively limited number of studies on hemorrhagic stroke restricts the robustness of conclusions. Future studies using standardized methodologies to define and measure TyG levels and stroke outcomes must confirm the observed associations.

Moreover, additional research should investigate the biological mechanisms that explain the relationship between elevated TyG levels and poor stroke outcomes. Furthermore, investigations are needed to determine whether interventions targeting TyG levels, such as lifestyle modifications or pharmacological therapies, can improve stroke prognosis. Lastly, studies incorporating cost-effectiveness analyses and evaluating the feasibility of integrating TyG index assessments into routine clinical practice could help guide its application in stroke prevention and management.

## CONCLUSION

Elevated TyG index might be a significant predictor of mortality, recurrence, END and poor functional outcomes in stroke patients, further emphasizing the potential of the TyG index as a prognostic biomarker. The TyG index shows potential as a tool for early risk assessment and tailored management in stroke care. However, further research is warranted to address current limitations and expand its clinical utility.

### Authors’ contributions:

**YZ and YC:** Literature search, study design and manuscript writing.

**YZ, YC and JW:** Data collection, data analysis and interpretation. Critical Review.

**YZ and YC:** Manuscript revision and validation and is responsible for the integrity of the study.

All authors have read and approved the final manuscript.
